# Appraisal Resources in an Academic Genre: English versus Persian Nutrition Research Articles

**DOI:** 10.1155/2021/6659796

**Published:** 2021-02-25

**Authors:** Mavadat Saidi

**Affiliations:** Shahid Rajaee Teacher Training University, Tehran, Iran

## Abstract

Academic study writers utilize a specific set of discursive resources backed up by both cultural and professional norms to be endorsed by the intended audience. The current study aimed to investigate if there were any significant differences between English and Persian academic research articles published in peer-reviewed journals in the field of nutrition in light of appraisal theory. To this end, a corpus of 40 English and 40 Persian academic research articles was analyzed in terms of three categories and subcategories of appraisal theory. The results revealed that the authors of both English and Persian research articles included more attitude resources followed by graduation and engagement resources. Furthermore, no significant difference was observed between these two sets of articles in terms of three major appraisal categories. The findings carry pedagogical implications for English for academic purposes courses to provide the academic discourse communities with the required discursive competencies.

## 1. Introduction

Academic articles such as “the main vehicles … to make new knowledge” ([1], p. 54) are read by the professional group of audience and seem to require a specific set of discursive resources to be well endorsed and understood by the intended audience [2]. As the predominant academic genre, RAs present “a credible account” of academics and enable them to express their solidarity with other specialists, gauge the coming ideas, and embrace viable alternatives ([3], p. 116). One of the tools which would enable scientists to interact more effectively with their audience is appraisal resources. Indeed, academic writers try “to inform and persuade readers of the truth of their statements” (Harris, 1991, p. 289) by adopting evaluative language resources [4], which are represented in the categories and subcategories of appraisal theory.

There has been a large body of research focusing on evaluation in academic discourse. Different academic genres have been examined in light of appraisal theory including RAs [5–7], RA abstracts [8, 9], RA introductions [10], RA conclusions [11], referee reports [12], and academic book reviews [13, 14].

On the other hand, various norms and expectations of a cultural community along with those of a particular academic community seem to play a key role in mediating the relationship between the writer and the reader [11, 15, 16]. Writers follow some cultural as well as professional norms when they present their arguments and engage with their intended audience [17]. There are cultural variations regarding the conventions for scientific and academic writing [15, 16]. This seems to justify conducting a contrastive study of RAs to see how authors of different cultural backgrounds lead their audience through the texts. As such, numerous studies have addressed the possible cross-cultural variation of various academic and scientific genres, for instance, RA conclusions [18], introduction, discussion, and conclusion sections of RAs [19–21], theses [22], and book reviews [23] across different languages such as English and Spanish [18, 20, 22], English and Finnish [24, 25], English and Japanese [23], English and Chinese [19], and English and Bulgarian [21]. A large number of studies have also aimed to shed more light on the possible cross-cultural variation of the academic genre across English and Persian languages [26–28] and mostly addressed the use of metadiscourse markers.

With this in mind, the current study aimed to analyze the English and Persian academic RAs in the field of nutrition employing appraisal theory to unfold the interpersonal meanings embedded in academic discourse [29] from a cross-cultural perspective.

## 2. Literature Review

Appraisal theory scrutinizes the embedded interpersonal meaning in a text. According to White [30], it entails “an approach to exploring, describing, and explaining the way language is used to evaluate, to adopt a stance, to construct textual personas, and to manage interpersonal positioning and relationships” (p. 1). It relies on previous works on evaluation [4] and is primarily related to the concept of evaluation defined by Hunston and Thompson ([31], p. 5) as “a broad cover term for the expression of the writer's attitude or stance towards, viewpoint on, or feelings about entities or propositions that he or she is talking about.”

Appraisal theory allows for a thorough exploration of evaluation typified by a set of syntactic-semantic resources [10, 32]. It encompasses three main domains, attitude, engagement, and graduation [4]. Attitude resources, which represent feelings, include affect, judgment, and appreciation: (1) affect entails a set of resources for shaping emotional reactions (e.g., I am sad, I hate chocolate) [4], (2) judgment is concerned with resources for admiring or criticizing behaviors (e.g., corruptly, skillfully) [32], and (3) appreciation encompasses resources for evaluating entities (e.g., beautifully, striking) [32].

Engagement provides resources for considering alternative positions and including heteroglossic variations (e.g., perhaps, it seems, I declare, and however) called heterogloss [4] and presenting absolute ideas or presuming a conversion between the sender and recipient of a message called monogloss [33].

Graduation encompasses lexicogrammatical resources used to strengthen or soften the attitudes [32]. In this sense, force entails resources for “grading according to intensity or amount” (e.g., a true friend) and focus resources for “grading according to prototypicality and the preciseness by which category boundaries are drawn” (e.g., kind'v, sort'v, as good as) ([4], p. 137).

A myriad of research has been conducted to unravel the representation of appraisal resources in various genres. Stotesbury [9] explored evaluation in RA abstracts and discovered cross-disciplinary variations considering the number and type of explicit evaluations. Moreover, Hyland and Tse [8] investigated number of times the evaluative “that” was employed in 405 abstracts and analyzed its various functions throughout the text. Furthermore, Gallardo and Ferrari [34] examined the appraisal resources in a discussion forum of doctors and found out the frequent application of negative attitude markers.

Also, Babaii [13] unfolded the use of personal comments, mockery, sarcasm, and unhedged and blunt criticism in book reviews in the field of physics. More recently, author (2017) tried to explore the evaluative resources of appraisal theory in popular science articles. The findings revealed that scientists took advantage of attitude resources more frequently followed by graduation and engagement resources when they addressed the nonscholarly audience.

Despite the huge bulk of research on appraisal resources across numerous genres, an obvious gap seems to exist considering the possible commonalities and discrepancies between English and Persian academic research articles in terms of the frequency of evaluative resources of appraisal framework to see how authors attempt “to inform and persuade readers of the truth of their statements” (Harris, 1991, p. 289).

## 3. Method

The corpus of the study consisted of 80 academic research articles (40 English and 40 Persian academic research articles) comprising a total of 141378 words (88561 words in English academic research articles and 52817 words in Persian academic research articles).

First, a comprehensive list of professional journals in the field of nutrition was collected from the following databases: www.journals.cambridge.org, www.online.sagepub.com, www.sceincedirect.com, and www.en.wikipedia.org/wiki/List_of_sceintific_journals≠Nutrition. Then, four experienced and currently active associate professors (three Iranian and one American one) in the field of nutrition and five Ph.D. students with research experience of more than three years were asked to provide expert judgment on the list of journals along with their impact factors. They were asked to add any other journal which was of high impact factor in the field if it was not included in the prepared list. The common professional sources were selected, and the new list was given to 2 associate professors and 5 Ph.D. students of nutrition to be evaluated for the last time. They were asked to rank the journals included in the list. Four English professional journals and two Persian professional journals were chosen for sampling academic sources. It is worth noting that the academic journals with the highest-ranking were selected taking into account both their ranking by experts in the prepared list and their impact factor values.

The ultimate selected English professional journals were Public Health Nutrition, The Journal of Nutrition, American Journal of Clinical Nutrition, and European Journal of Clinical Nutrition.

The ultimate selected Persian professional journals were Oloom-e-Taghziye-va-Sanaye-Ghazaie-e-Iran (Iranian Journal of Nutrition Sciences and Food Technology) and Oloom-e-Ghazaie-va-Taghziye (Journal of Food Technology and Nutrition).

Iranian Journal of Nutrition Sciences and Food Technology is an official scientific quarterly publication of the National Nutrition and Food Technology Research Institute (NNFTRI) and publishes original research, review articles, short communications, and letters to the editor which have not been previously published. It is indexed in Chemical Abstracts Service, CINAHL, Index Copernicus, EMRO, CABI, ISC, Iran Medex, and Magiran. It is accessible from http://nsft.sbmu.ac.ir.

Journal of Food Technology and Nutrition is a scientific quarterly that publishes original articles in the field of food sciences, nutrition, and chemical engineering. It is indexed in SID, ISC, and Magiran. It is affiliated with Islamic Azad University, Science and Research branch. It is accessible from http://jftn.srbiau.ac.ir.

Accordingly, ten articles published from 2010 to 2015 were selected from the archive of each academic source. The Persian academic research articles were written by Persian native speakers. Considering the English academic research articles, the researcher chose those articles which had at least one author with a European or American affiliation. Academic research articles in medical fields of study mostly have more than one author due to complicated experimental procedures. Hence, academic research articles were not single-authored. As such, it seemed impossible to ensure that the authors were native speakers of English. However, since the journal from which the articles were selected was highly prestigious and ranked internationally in the field of nutrition, they surely undergo a very precise publication process, and it seems necessary for the authors to be acquainted with English academic conventions [20]. Moreover, the members of the editorial and advisory board were mostly both native speakers and professional experts in the field. These all seem to guarantee the precision of language and supervision over the quality of the articles.

Only the “Results” and “Discussion” sections of the academic research articles were considered. As Fahnestock [35] maintained, “Results” and “Discussion” sections of academic research articles are “the best possible representation for the physical evidence the researcher generated” (p. 333). These two sections establish the validity of the findings and report the outcomes of a scientific procedure and the possible reasons justifying them.

Appraisal framework “as a system of semantic resources” ([32], p. 74) unravel “the array of interpersonal resources variously concerned with authorial attitude, social evaluation, and positioning of both reader and authorial voice” (p. 75).

First, the cases of appraisal resources were coded in each article. Following SFL, the unit of analysis was clauses [13].

Then, two MA graduates of applied linguistics analyzed all the articles in the corpus, and an intercoder reliability coefficient of 0.91 was obtained. They were familiar with the appraisal theory and its categories. Then, all categories (attitude, engagement, and graduation) and subcategories (affect, appreciation, judgment; monogloss, heterogloss; and force, focus) of appraisal resources were counted.

The frequency and percentage values were reported. Then, in order to compare the academic research articles of various lengths, the raw frequencies were normalized to 1000 words [36]. For doing so, each raw frequency is divided by the number of words in that corpus and multiplied by 1000 [37]. Since the data were nominal, several statistical nonparametric tests of chi-square were conducted to see if there were any significant differences between English and Persian academic research articles in terms of appraisal resources.

The current study adopted one of the mixed-methods designs named conversion design which involves “transforming data” ([38], p. 563). According to Ary et al. [38], in conversion designs, “qualitative data might be quantitized by counting … the number of times a particular theme is identified” (p. 564). When the researcher begins with qualitative data and analysis and transforms the words into numerical values for conducting a statistical and comparative analysis, the study entails a conversion design.

## 4. Results and Discussion

The study aimed to explore the frequency of appraisal resources in English and Persian academic research studies published in four leading English and two leading Persian journals which addressed coding attitude (affect, appreciation, and judgment), engagement (monogloss and heterogloss), and graduation (force and focus) resources.  Table 1 presents the frequency counts that display how appraisal resources were distributed in English and Persian academic research articles.

The results of the analysis of English academic research articles in terms of appraisal resources revealed that authors included more attitude resources. Out of 4744 identified appraisal resources, 2956 (62.30%) were attitude resources in comparison with 462 (9.75%) engagement resources and 1326 (27.95%) graduation resources. Similarly, the results of the analysis of Persian academic research articles in terms of appraisal resources revealed that authors used more attitude resources. Out of 3126 identified appraisal resources, 1848 (59.10%) were attitude resources in comparison with 530 (16.95%) engagement resources and 748 (23.95%) graduation resources.

As Table 2 shows, no significant difference was found between English and Persian academic research articles in terms of attitude resources of appraisal theory.

Similarly, no significant difference existed between English and Persian academic research articles considering both engagement (Table 3) and graduation (Table 4) resources of appraisal theory.

Among attitude markers, the authors of both English and Persian academic research articles employed appreciation resources (2947 (99.69%) and 1848 (100%), respectively) more frequently than the other two subcategories. In this regard, the authors used appreciation resources to attribute some values to the things and presented an evaluative language to describe the natural phenomena and entities. The appreciation resources were used to reflect the authors' positive or negative feelings towards products (e.g., healthy bed, rich in leucine, and rich source), processes (e.g., challenging issue, negligible change, unhealthy lifestyle, in/significant impact, important role, and strict mechanism), and entities (e.g., nutritious food source, poor pattern, and notable point) [32].

Following abundant appreciation resources, the authors of English academic research articles included few cases of affect resources (8 (0.27%), respectively). Contrarily, no cases of affect resources were coded in Persian academic research articles.

1. Mothers who are worried about the quality of their child's diet (European Journal of Clinical Nutrition, 2010)
2. Some consumers who are concerned about “food miles” and the carbon emissions (Public Health Nutrition, 2012)
3. That could help parents feel good about the way they feed their families (Public Health Nutrition, 2011)
4. In the above examples, the affect resources were represented in the form of “mental processes of reaction” (examples 1 and 3) and “attributive relationals of affect” (example 2) to reflect how the authors attributed positive and negative feelings to individuals ([32], p. 75)
5. The least frequently used category of attitude resources in English academic research articles was judgment (1 (0.04%)), while Persian academic research articles included no judgment resources
6. This seems to be in accordance with consumers' perception that ready meals and fast food are not seen as appropriate for dinner meals (Public Health Nutrition, 2011)
7. In example 4, the author confirmed the consumers' perception and expressed a positive evaluation by referring to its accordance with the results of the given study
8. Considering the number of graduation resources, English academic research articles encompassed 1322 force resources (99.70%) and only 4 focus resources (0.30%)
9. Ranging from 42% in the least disadvantaged areas to 43% in the most disadvantaged areas (Public Health Nutrition, 2012)
10. Arginine and lysine were not very effective in activating the CaSR
11. Higher calcium and phosphorus intakes could have masked any potential (The Journal of Nutrition, 2014)
12. “Restriction” was the only feeding group (European Journal of Clinical Nutrition, 2010)
13. Compared with approximately one-third of children whose mother did not (American Journal of Clinical Nutrition, 2013)
14. In the above examples, the authors of English academic research articles employed force resources (e.g., the least, the most, -er, very, only, approximately) to either strengthen or mitigate their appreciation [32] to show the extent of the characteristic they attributed to a process, product, and entity.
15. However, only 4 (0.30%) focus resources were included in English academic research articles
16. Although red meat is an excellent source of iron (American Journal of Clinical Nutrition, 2010)
17. …If there is a genuine birth cohort effort (Public Health Nutrition, 2010)
18. …Half glass of juice is really 100% juice (Public Health Nutrition, 2011)
19. In these examples, the authors tried to sharpen the focus and expressed the preciseness of the existence of an attribute [32]
20. On the other hand, the Persian academic research articles included only 748 force resources (100%), and no focus resources were coded in the Persian academic research sample. In these examples, force resources (e.g., the most frequent, very effective, approximately natural, more powerful drive, and about half of) were used to show the authors' tendency for increasing or decreasing the degree of appreciation of a process, product, and entity [32].
21. With regard to the engagement category, the authors of both English and Persian academic research articles included more heterogloss (462 (100%) and 520 (98.10%), respectively). On the other hand, while the authors of English academic research articles included no monogloss in their communication of scientific facts about nutrition, there were few cases of monogloss (10 (1.90%)) in their Persian counterparts'.
22. The present study supports findings by Lachat et al. (Public Health Nutrition, 2011)
23. Several additional studies reporting indispensable amino acid first pass
24. One study met all methodological criteria as described previously (The Journal of Nutrition, 2013)
25. Also consistent with the previous work, maternal obesity (American Journal of Clinical Nutrition, 2013)
26. …But some support comes from longitudinal children in which (European Journal of Clinical Nutrition, 2010)
27. In contrast to the study by Dawson-Hughes et al., our baseline dietary (The Journal of Nutrition, 2014)

In the above examples, the authors of English academic research articles referred to the findings of previous studies (examples 13, 14, 15, and 16) or some alternative ideas (example 17). Similarly, the Iranian authors highlighted the results of available research (e.g., the results were in line with those of Goutam in 2009, few studies have shown that) or acknowledged other possible ideas (e.g., it is believed that, one of the possible factors, the other method for).

There were 10 cases (1.9%) of monogloss in Persian academic research articles sample in which the authors presented a scientific fact without citing a reliable source.

The results of the analysis of both English and Persian academic research articles in terms of appraisal resources revealed that the authors of both groups of academic research articles had a higher preference to employ attitude resources and embedded their feelings in the texts [4]. Among attitude subcategories, appreciation resources were the most frequently used ones by which the authors aimed to build in values for the findings ([32], p. 36) and evaluate products, processes, and entities. Following the appreciation subcategory of attitude resources, the authors of English academic research articles included few affect resources. While no case of these two subcategories (affect and judgment) was observed in Persian academic research articles, only one case of judgment was coded in English academic research articles. Moreover, the results of chi-square tests revealed that there was no significant difference between English and Persian academic research articles in terms of three main categories of appraisal resources, i.e., attitude, engagement, and graduation resources.

Although academic writing has been considered as “dispassionate description of truth” (Penrose and Katz, 1998, p. 169, cited in [39], p. 9) for a long time, academic writers inevitably seek both to inform and to persuade readers of the truth value of their claims [8, 39]. So, they tend to both “involve themselves in the written communication” and observe “the objective or impersonal convention of the academic community” ([39], p. 9). In line with those of studies which revealed the use of interpersonal resources in academic writing [39], the results of the current study proved that attitude resources, especially appreciation resources, were included to invoke “academic persuasion” (Hyland, 2008, p. 2, cited in [39], p. 10).

The scientists seem to take advantage of evaluative resources to convince their audience of the validity of their recent scientific discoveries (Miller, 1998). It might be said that even the scientists try to influence other scientists through employing such resources as appreciation. However, this seems to be performed based on a reasonably consistent set of standards and conventions. They are simultaneously engaged in the process of “establishing factual information” rather than “overtly providing value judgments” (Herriman, 200b, cited in [39], p. 10).

The scientists' tendency to persuade and convince their audience as well as their intention to stick to the norms, standards, and conventions of the scientific discourse community lead to including more cases of appreciation resources and few, and even no, cases of affect and judgment. It seems that scientists are aware of the power of appealing to “media rules” rather than the strict “institutional values of science” ([1], p. 173) for scientific facts to be embraced even by the scientific discourse community. As Hyland and Tse [8] rightly mentioned, academic arguments need “subjective judgments” and “interpretive statements” to draw the other scientists' attention. Simultaneously, academic research articles should be structured [40] and depict “scientific activity as a set of procedures designed to test experimental validity” ([41], p. 755).

Moreover, making use of attitude resources in general and appreciation resources in particular, on the one hand, might point to the “groundlessness of the myth that views professional biological writing as consisting only of impersonal, factual statements” ([42], p. 118). On the other hand, the point that affect and judgment subcategories were rarely (never, in the case of Persian sample) used in academic research articles might be attributed to the scientists' tendency to convince their audience that the research findings result from objective observations of a natural phenomenon rather than their subjective beliefs. Scientists never provide a negative judgment of their ideas or those of other scientists to make an impression on the intended audience. Nevertheless, they seem to use the safest subcategory of attitude resources, i.e., appreciation resources, to present an evaluation of products, processes, and entities rather than judging or attributing a characteristic to human participants.

In addition to attitude resources of appraisal theory, the authors of both English and Persian academic research articles used graduation resources. Both English and Persian academic research articles used force resources more frequently while few focus resources were coded only in English academic research articles. It is natural in scientific articles to present the results cautiously rather than claiming absolute truth [8, 43]. Indeed, scientists are inclined to stick to academic community norms, one of which is to avoid overgeneralization and to look at their findings as some probable phenomena, instead [8].

The least frequently used category of appraisal resources was engagement. As was expected, the authors made use of more heterogloss resources than those of monogloss, and only few cases of monogloss resources, which seem negligible, were coded in Persian academic research articles. As Gallardo [44] demonstrated, scientists cite other scientists, mostly more prominent figures in the field, to manage their arguments about their recent scientific findings and convey their intended message. In this way, they try to follow the key to succeed by showing solidarity with the community to which they belong and respecting the common goals and conventions [11, 45]. Using heterogloss resources, scientists are enabled to “surpass their personal perspectives” and keep the authoritative function of science ([45], p. 389).

On the other hand, since writer and reader seem to have unequal power relations, i.e., the writer addresses the research community as the reader of academic research articles, and thereby, convincing the audience appears to be much challenging [45]. In this sense, scientists prefer to base their arguments on previously proven findings.

In contrast with previously conducted studies in which English authors were shown to employ a higher or lower number of metadiscourse markers than those of other languages [24, 26], Duszak, 1994 [19, 23], the results of the current study revealed that both English and Persian authors made equal use of appraisal resources. This might suggest that Persian authors follow the norms of the academic discourse community of nutrition on the international scale. This seems rational as the intended audience of these two Persian professional journals consists of experts in the field of nutrition who are quite familiar with the rules of the nutrition games. It may also point to the authors' awareness of the necessity of being heard internationally in the era of globalization [46]. Hence, it might be concluded that the value and belief systems of the academic discourse community of nutrition are quite the same among Persian and English experts in the field who address their counterparts in the report of their recent discoveries.

## 5. Conclusion and Implications

The study tried to explore the frequency of appraisal resources across English and Persian academic research articles. The findings indicated that the authors of both English and Persian research articles took more advantage of attitude resources followed by graduation and engagement ones. Moreover, no significant difference was found between English and Persian academic research studies in terms of three categories of appraisal resources. The results indicated that despite the common belief about the objective or impersonal nature of the academic research articles, even scientists have come to this understanding that employing attitude resources would enhance the persuasiveness of this genre [39]. Nevertheless, scientists still stick to the academic norms of the community by appealing to the findings of previous studies and allowing for alternative stances utilizing a higher number of heteroglossic and force resources.

The findings of the current study would enrich the available literature on the discursive practices in academic genres and carry pedagogical implications. The findings would benefit the English for Academic Purposes (EAP) writing instructors and applicants through familiarizing them with the current discursive practices that could persuade their audience to embrace their discoveries. In this sense, EAP writing courses could set the scene for the would-be-scientists “to learn the skills needed to address the readers, both in terms of content choices and modes of presentation” ([2], p. 3). Furthermore, as globalization necessitates “being heard internationally” ([46], p. 292), the findings would raise the EAP learners' consciousness about the persuasive tools they can utilize to accommodate their scientific discoveries in the international scientific community [47].

The present study also gives rise to more research questions considering the frequency of other linguistic and pragmatic features in academic research articles in other fields of study across various languages. Moreover, appraisal resources can be investigated in the oral mode of academic genres, namely, conference presentations. Furthermore, appraisal resources can be taught to EAP course applicants to see if their perception and production of academic research articles can be influenced.

## Figures and Tables

**Table 1:** Frequencies of appraisal resources in English and Persian academic research articles.

Appraisal resources	Total frequency		Normalized frequency	
	English	Persian	English	Persian
Attitude	2956	1848	333.78	349.88
Affect	8	0	0.90	0
Appreciation	2947	1848	332.76	349.88
Judgment	1	0	0.11	0
Engagement	462	530	52.16	98.45
Monogloss	0	10	0	1.89
Heterogloss	462	520	52.16	98.45
Graduation	1326	748	149.72	141.62
Force	1322	748	149.72	141.62
Focus	4	0	0.45	0
Total	4744	3126	535.67	591.85

**Table 2:** Chi-square test for attitude resources across English and Persian academic research articles.

	Value	df	Significance
English vs. Persian academic research articles	1.049	2	0.592

**Table 3:** Chi-square test for engagement resources across English and Persian academic research articles.

	Value	df	Significance
English vs. Persian academic research articles	1.054	1	0.305

**Table 4:** Chi-square test for graduation resources across English and Persian academic research articles.

	Value	df	Significance
English vs. Persian academic research articles	0.000	1	1.000

## Data Availability

The data used to support the findings of this study are available from the corresponding author upon request.

## References

[1] Russell N. (2010). *Communicating Science*.

[2] Bowler P. J. (2009). *Science for All: The Popularization of Science in Early Twentieth-Century Britain*.

[3] Hyland K. (2010). Constructing proximity: relating to readers in popular and professional science. *Journal of English for Academic Purposes*.

[4] Martin J. R., White P. R. P. (2005). *The Language of Evaluation: Appraisal in English*.

[5] Dueñas P. M. (2010). Attitude markers in business management research articles: a cross-cultural corpus-driven approach. *International Journal of Applied Linguistics*.

[6] Thetela P. (1997). Evaluated entities and parameters of value in academic research articles. *English for Specific Purposes*.

[7] Tutin A., Lores-Sanz R., Mur-Duenas P., Laufauente-Millan E. (2010). Evaluative adjectives in academic writing in the humanities and social sciences. *Constructing Interpersonality: Multiple Perspectives on Written Academic Genres*.

[8] Hyland K., Tse P. (2005). Hooking the reader: a corpus study of evaluative *that* in abstracts. *English for Specific Purposes*.

[9] Stotesbury H. (2003). Evaluation in research article abstracts in the narrative and hard sciences. *Journal of English for Academic Purposes*.

[10] Hood S. (2004). *Appraising Research: Taking a Stance in Academic Writing*.

[11] Abdollahzade E. (2011). Poring over the findings: interpersonal authorial engagement in applied linguistics papers. *Journal of Pragmatics*.

[12] Fortanet I. (2008). Evaluative language in peer review referee reports. *Journal of English for Academic Purposes*.

[13] Babaii E., Salager-Mayer F., Lewin B. A. (2011). Hard science, hard talk? The study of negative comments in physics book reviews. *Crossed Words, Criticism in Scholarly Writing*.

[14] Moreno A. I., Suárez L. (2008). A framework for comparing evaluation resources across academic texts. *Text and Talk*.

[15] Clyne M., Shroder H. (1991). The socio-cultural dimension: the dilemma of the German-speaking scholar. *Subject-Oriented Texts: Language for Special Purposes and Text Theory*.

[16] Galtung J., Gakung J. (1979). Deductive thinking and political practice: an essay of the teutonic intellectual style. *Essays on Methodology*.

[17] Kaplan R. B., Silva T., Matsuda P. K. (1966). Cultural thought patterns in inter-cultural education. *Landmark Essays on ESL Writing*.

[18] Moreno A. I. (1997). Genre constraints across languages: causal metatext in Spanish and English RAs. *English for Specific Purposes*.

[19] Hu G., Cao F. (2011). Hedging and boosting in abstracts of applied linguistics articles: a comparative study of English- and Chinese-medium journals. *Journal of Pragmatics*.

[20] Mur-Dueñas P. (2011). An intercultural analysis of metadiscourse features in research articles written in English and in Spanish. *Journal of Pragmatics*.

[21] Vassileva I. (2001). Commitment and detachment in English and Bulgarian academic writing. *English for Specific Purposes*.

[22] Soler-Monreal C., Carbonell-Olivares M., Gil-Salom L. (2011). A contrastive study of the rhetorical organisation of English and Spanish PhD thesis introductions. *English for Specific Purposes*.

[23] Itakura H. (2013). Hedging praise in English and Japanese book reviews. *Journal of Pragmatics*.

[24] Crismore A., Markkanen R., Steffensen M. S. (1993). Metadiscourse in persuasive writing. *Written Communication*.

[25] Mauranen A. (1993). Contrastive ESP rhetoric: metatext in Finnish-English economics texts. *English for Specific Purposes*.

[26] Abdollahzadeh E. Interpersonal metadiscourse in ELT papers by Iranian and anglo-American academic writers.

[27] Marandi S. (2003). Metadiscourse in Persian and English master’s thesis: a contrastive study. *Iranian Journal of Applied Linguistics*.

[28] Zarei GH. R., Mansoori S. (2009). Metadiscourse in academic prose: a contrastive analysis of English and Persian research articles. *The Asian ESP Journal*.

[29] Wei Y., Wherrity M., Zhang Y. (2015). An analysis of current research on the Appraisal Theory. *Linguistics and Literature Studies*.

[30] White P. P. R. (2014). http://www.grammatics.com/appraisal/AppraisalGuide.

[31] Hunston S., Thompson G. (2000). *Education in Text: Authorial Stance and the Construction of Discourse*.

[32] White P. R. R. (1998). *Telling Media Tales: The News Story as rhetoric*.

[33] Lee S. H. (2006). *The Use of Interpersonal Resources in Argumentative/persuasive Essays by East-Asian ESL and Australian Tertiary Students*.

[34] Gallardo S., Ferrari L. (2010). How doctors view their health and professional practice: an appraisal analysis of medical discourse. *Journal of Pragmatics*.

[35] Fahnestock J. (1986/1998). Accommodating science: The rhetorical life of scientific facts. Written Communication. *Reprinted in Written Communication*.

[36] Biber D., Conrad S., Reppen R. (1998). *Corpus Linguistics: Investigating Language Structure and Use*.

[37] Aktas R. N., Cortes V. (2008). Shell nouns as cohesive devices in published and ESL student writing. *Journal of English for Academic Purposes*.

[38] Ary D., Jacobs L. Ch., Sorensen Ch. K. (2010). *Introduction to Research in Education*.

[39] Zhang G. (2015). It is suggested that…or it is better to…? Forms and meanings of subject it-extraposition in academic and popular writing. *Journal Of English for Academic Purposes*.

[40] Hempel S., Degand L. (2008). Sequencers in different text genres: academic writing, journalese and fiction. *Journal of Pragmatics*.

[41] Corbett J., Asher R. E., Simpson J. M. (2006). Popularizations. *Encyclopedia of Language and Linguistics*.

[42] Crismore A., Farnsworth R., Nash W. (1990). Metadiscourse in popular and professional science discourse. *The Writing Scholar: Studies in Academic Discourse*.

[43] Varttala T. (1999). Remarks on the communicative functions of hedging in popular scientific and specialist research articles on medicine. *English for Specific Purposes*.

[44] Gallardo S. (2005). Pragmatic support of medical recommendations in popularized texts. *Journal of Pragmatics*.

[45] Parkinson J., Adendorff R. (2004). The use of popular science articles in teaching scientific literacy. *English for Specific Purposes*.

[46] Shaw P., Vassileva I. (2009). Co-evolving academic rhetoric across culture; Britain, Bulgaria, Denmark, Germany in the 20th century. *Journal of Pragmatics*.

[47] Dafouz-Milne E. (2008). The pragmatic role of textual and interpersonal metadiscourse markers in the construction and attainment of persuasion: a cross-linguistic study of newspaper discourse. *Journal of Pragmatics*.

